# Spatial accuracy of dose delivery significantly impacts the planning target volume margin in linear accelerator-based intracranial stereotactic radiosurgery

**DOI:** 10.1038/s41598-025-87769-z

**Published:** 2025-01-29

**Authors:** Yuta Takahashi, Riki Oshika, Rie Tachibana, Katsuyuki Shirai, Hiroshi Asakura, Masayoshi Miyazaki, Tomohiro Sagawa, Shinichi Takahashi, Tsunekazu Kuwae, Hironori Kojima, Shiro Nishiyama, Hikaru Nemoto, Yoshitomo Ishihara, Mariko Umeda, Kotaro Kijima, Daisuke Kobayashi, Keiji Suzuki, Yuki Nozawa, Kento Hoshida, Tomoki Kitagawa, Hiromitsu Endo, Yuki Matsunaga, Hiroya Itagaki, Mayumi Ishida, Shigeru Kanahara, Ryo Horita, Daisuke Hori, Hidenobu Tachibana

**Affiliations:** 1 Division of Radiation Medical Physics, Jichi Medical University Saitama Medical Center, Saitama, Japan; 2 Radiation Safety and Quality Assurance division, National Cancer Center Hospital East, Chiba, 277-8577 Japan; 3 Triangle Products Co. Ltd, Chiba, Japan; 4 Department of Radiation Oncology, Jichi Medical University Hospital, Tochigi, Japan; 5 Radiation Oncology Center, Dokkyo Medical University Hospital, Tochigi, Japan; 6 Department of Radiation Oncology, Osaka International Cancer Institute, Osaka, Japan; 7 Division of Radiation Technology, Hospital East, National Cancer Center, Chiba, Japan; 8 Division of Radiology, Yuuai Medical Center, Okinawa, Japan; 9 Department of Radiology, Kanazawa University Hospital, Kanazawa, Ishikawa Japan; 10 Department of Radiotechnology, Saiseikai Kawaguchi General Hospital, Saitama, Japan; 11 Department of Radiology, University of Yamanashi, Yamanashi, Japan; 12 Department of Radiation Oncology, Division of Medical Physics, Japanese Red Cross Wakayama Medical Center, Wakayama, Japan; 13 Department of Radiation Oncology, Saitama Medical Center, Saitama Medical University, Saitama, Japan; 14 Department of Radiology, NHO Saitama Hospital, Saitama, Japan; 15 Department of Radiology, University of Tsukuba Hospital, Ibaraki, Japan; 16 Department of Radiology, The University of Tokyo Hospital, Tokyo, Japan; 17 Department of Radiology, Kurume University Hospital, Fukuoka, Japan; 18 Department of Radiation Oncology, Aichi Cancer Center Hospital, Nagoya, Aichi Japan; 19 Department of Radiation Physics and Technology, Southern TOHOKU General Hospital, Fukushima, Japan; 20 Department of Radiology, Fukuoka Tokushukai Hospital, Fukuoka, Japan; 21 Department of Radiology, Niigata City General Hospital, Niigata, Japan; 22 Division of Radiology, JCHO Osaka Hospital, Osaka, Japan; 23 Central Radiology Division, Kawasaki Medical School General Medical Center, Okayama, Japan; 24 Central Radiology Division, Nagoya City University East Medical Center, Aichi, Japan; 25 Department of Radiology, Japanese Red Cross Nagasaki Genbaku Hospital, Nagasaki, Japan

**Keywords:** SRS, Linac, Three-dimensional dose delivery accuracy, Margin size, Gel dosimeter, Multi-institution, Radiotherapy, CNS cancer

## Abstract

The impact of three-dimensional (3D) dose delivery accuracy of C-arm linacs on the planning target volume (PTV) margin was evaluated for non-coplanar intracranial stereotactic radiosurgery (SRS). A multi-institutional 3D starshot test using beams from seven directions was conducted at 22 clinics using Varian and Elekta linacs with X-ray CT-based polymer gel dosimeters. Variability in dose delivery accuracy was observed, with the distance between the imaging isocenter and each beam exceeding 1 mm at one institution for Varian and nine institutions for Elekta. The calculated PTV margins for Varian and Elekta linacs that could cover the gross tumor volume with 95% probability at 95% of the institutions were 2.3 and 3.5 mm, respectively, in the superior–inferior direction. However, with multifactorial system management (i.e., high-accuracy 3D dose delivery with rigorous linac quality assurance, strict patient immobilization, and high intra-fractional positioning accuracy), these margins could be reduced to 1.0 mm and 1.5 mm, respectively. The findings indicate significant millimeter-level variability in 3D dose delivery accuracy among linacs installed in clinical settings. Thus, maximizing a linac’s 3D dose delivery accuracy is essential to achieve the required PTV margin in intracranial SRS.

**Supplementary Information:**

The online version contains supplementary material available at 10.1038/s41598-025-87769-z.

## Introduction

Stereotactic radiosurgery (SRS) and stereotactic radiotherapy (SRT) using a medical linear accelerator (linac) have largely replaced whole-brain irradiation as a treatment for multiple brain metastases^1–6^. For such applications, SRS and SRT commonly use non-coplanar, intensity-modulated irradiation^7,8^. The targets are often smaller than a few centimeters, and treatment is planned with a small planning target volume (PTV) margin of 0–2 mm^9–11^. This margin size is also recommended by the working group on stereotactic radiotherapy of the German Society of Radiation Oncology^12^. These facts highlight the need for radiation delivery with submillimeter accuracy. Minimizing irradiation positional errors relative to the imaging isocenter is therefore becoming increasingly important. Variations in linac dose delivery accuracy may also affect clinical outcomes of intracranial SRS/SRT. Several guidelines for SRS require a geometric accuracy within 1 mm in an end-to-end (E2E) test^13–15^. The E2E test includes the accuracy of contouring and registration^15^. Thus, the guidelines require the delivery accuracy of the linac to be less than 1 mm.

van Herk et al. designed a method to determine which uncertainties and what magnitudes of those uncertainties must be factored into the margin^16,17^. However, using systematic and random errors in patient positioning is common in clinical practices, and users rarely use the three-dimensional (3D) dose delivery accuracy of the linac^17^. In contrast, Takakura et al. suggested a method for estimating overall geometric uncertainty from uncertainties in both the patient position and linac dose delivery^18^. Moreover, Zhang et al. suggested a method to derive the optimal PTV margin for intracranial SRS by taking into account the distance between the imaging and radiation isocenters and the residual setup error after image guidance^19,20^. If the impact of the 3D dose delivery accuracy on the margin is high, the approach proposed by Zhang et al. and Takakura et al. is considered ideal. To correctly derive the margin, the 3D dose delivery accuracy of the linac must be measured with low measurement uncertainties.

To appropriately determine the margin for intracranial SRS/SRT and provide treatment with sufficient target coverage, we should directly assess and manage the 3D radiation delivery accuracy relative to the imaging isocenter by performing quality assurance (QA) procedures with minimal measurement uncertainties. Conventionally, two-dimensional (2D) planar images are obtained for each beam in QA tests, and the mechanical isocenter with some setup uncertainties is commonly used as the reference, especially for the Winston–Lutz (WL) test^15,21,22^. Thus, these tests include the measurement uncertainties derived from the mechanical isocenter to obtain 3D information from the 2D images. Uncertainties in a QA test may therefore contribute to the PTV margin size because the spatial dose delivery accuracy of linacs affects geometric uncertainties in the GTV.

Recently, a new 3D starshot (3D-SS) test using a polymer gel dosimeter has been reported to solve the above-mentioned problems in verifying geometric accuracy^23–25^. This approach allows a direct, comprehensive 3D evaluation of spatial radiation accuracy relative to the imaging isocenter^25^. The trajectories of radiation beams through the dosimeter are visualized using kilovoltage cone-beam computed tomography (kV-CBCT), and the imaging isocenter is thus obtained from the resulting kV-CBCT image. Human errors derived from QA phantom setup and laser indication adjustment do not affect the measurement accuracy. The 3D-SS test may be superior to the conventional QA methods because it eliminates uncertainties associated with those methods, and the measurement uncertainty of the 3D-SS test has been found to be 0.2–0.3 mm^25^.

The geometric uncertainties in patient setup and dose delivery may vary among institutions and manufacturers. The 3D dose delivery accuracy should be included in the PTV margin size if the uncertainties significantly affect the margin. This 3D dose delivery accuracy should be measured with low uncertainty, because the guidelines have recommended that each institution aim to achieve a PTV margin of 2 mm in intracranial SRS^12^. In contrast to conventional QA tests of spatial dose delivery, which may over- or underestimate the margin, 3D-SS analysis is effective for this purpose because it provides such spatial information free from measurement uncertainties associated with the setup. However, only the methodology has so far been proposed, and no information is available on variations in 3D dose delivery accuracy among commercially available linacs. While there are numerous studies focusing on patient setup accuracy^26–29^, there is a notable lack of research addressing linac precision, particularly with respect to differences between manufacturers and individual units. Furthermore, it remains unclear whether the PTV margin obtained for linac-based intracranial SRS via 3D-SS analysis will satisfy the recommended 2 mm limit^12^.

To correctly evaluate the impact of the spatial dose delivery accuracy of linacs on the PTV margin in intracranial SRS, we performed a multi-institutional study using linacs from two vendors using the 3D-SS test. We also developed a method for calculating the margin for the 3D-SS test. Furthermore, on the basis of the 3D-SS test results, we investigated whether the PTV margin could be constrained within 2 mm by implementing advanced image guidance (real-time video-based 3D optical surface imaging and 2D kV stereoscopic imaging) and rigorous linac quality management.

## Materials and methods

### Institution selection

Twenty-two institutions that performed intracranial stereotactic radiotherapy in clinical practices participated in this study. All institutions used a medical C-arm linac attached to an on-board imager enabling kV-CBCT. Seven institutions used a TrueBeam system (Varian Medical Systems, Inc., Palo Alto, CA, USA), four used a TrueBeamSTx system (Varian Medical Systems), four used a VersaHD system (Elekta AB, Stockholm, Sweden), three used an Infinity system (Elekta AB), and four used a Synergy system (Elekta AB). We verified that a geometric QA test was regularly performed at all facilities in accordance with guidelines published by task groups 142 and 198 of the American Association of Physicists in Medicine^21,22^.

### Assessment of 3D linac accuracy using the 3D-SS test

The 3D-SS test is robust with low measurement uncertainties for evaluating 3D dose delivery accuracy^25^. All institutions followed the 3D-SS test procedure described below. In total, 22 jars each containing an X-ray computed tomography (CT)-based polymer gel dosimeter called dGEL™ (Triangle Products Co., Ltd., Kashiwa, Japan) were used in this study^30^.

**Fig. 1:**
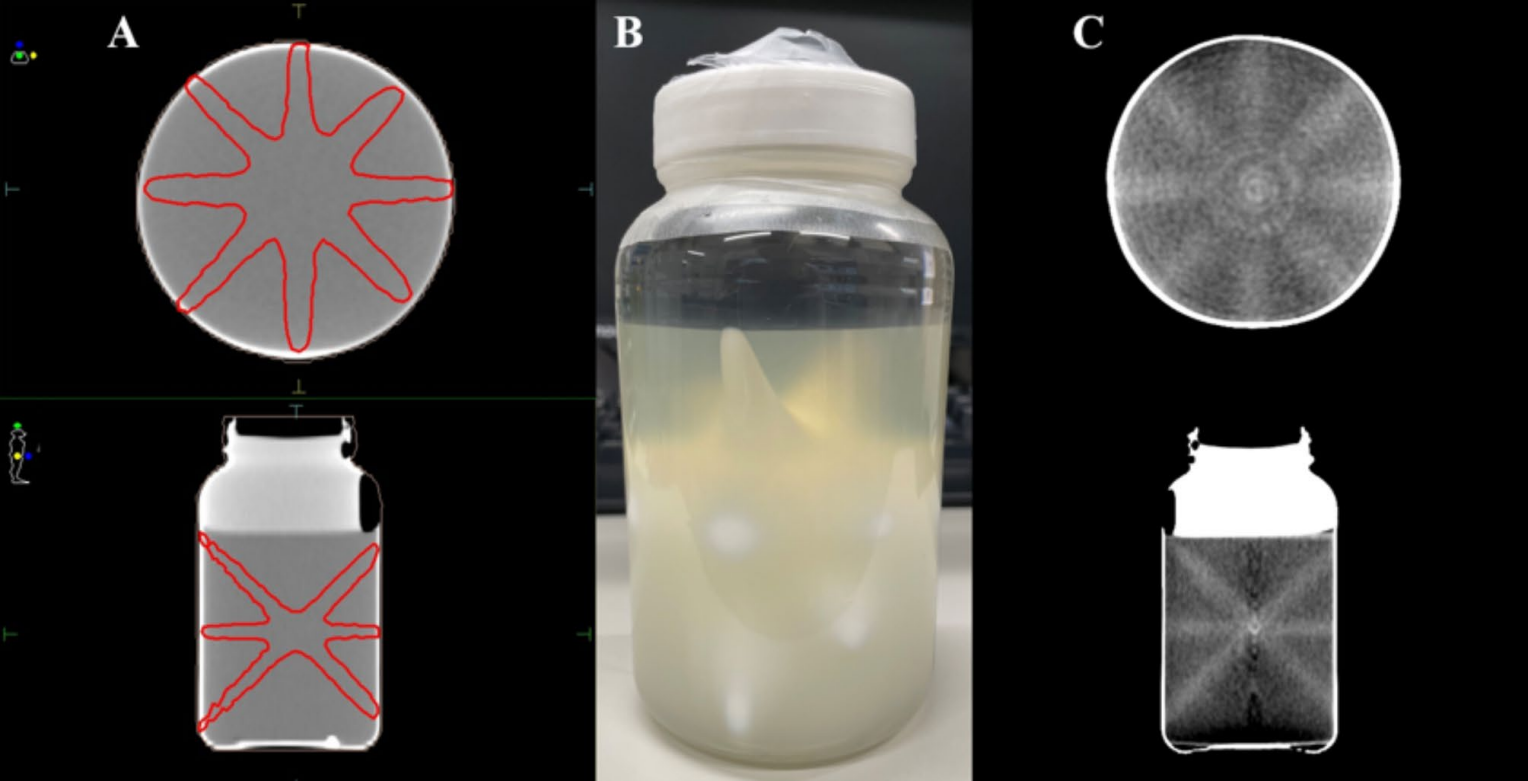
Three-dimensional starshot (3D-SS) treatment planning (**A**), irradiated gel dosimeter (**B**), and cone-beam computed tomography (CT) images of the post-irradiated gel dosimeter (**C**) used in this study. Red lines in panel A indicate the 20 Gy isodose line.

Treatment was planned using the treatment planning system installed at each institution. A common dGEL™ CT dataset, acquired using an Aquilion ONE system (Canon Medical Systems, Otawara, Japan), was sent to all institutions. A 10 MV flattening filter photon beam or a 6 or 10 MV flattening filter-free photon beam was used. All the Varian and Elekta systems used a 6 MV flattening filter photon beam to obtain the plot of the distance between the projected isocenter and the nominal isocenter for corrections of the mechanical errors of each CBCT system. Symmetric fields with a minimum size of 0.5 × 0.5 cm² for Varian systems and 0.6 × 0.6 cm² for Elekta systems were used. The field sizes of the Varian and Elekta systems were slightly different. Theoretically, these differences in field size do not affect the 3D-SS test results, because the beam axes are identified from the dose distributions by maximizing the contrast-to-noise ratio, with distance from the axis weighted appropriately during the optimization process^23,25^. Table 1 shows gantry, couch, and collimator angle settings for radiation beams based on the study by Pant et al.^23^ A machine output between 6000 and 8000 monitor units was determined for each beam to create an isodose line of 20 Gy in the beam path (Fig. 1A). A dose of 20 Gy was selected on the basis of our preliminary study^25^, which showed that this dose resulted in a sufficient contrast-to-noise ratio for each beam to perform the analysis using our in-house software. The software uses algorithms that determine a 3D vector with a point in the CBCT coordinate system that defines the beam axis by maximizing the contrast-to-noise ratio^25^. Each jar including the gel dosimeter was sent to its respective institution. The jar was placed with the center of its sensitive volume located approximately at the mechanical isocenter indicated by the room laser. The gel dosimeter was placed on the couch and secured in place using adhesive tape to prevent movement. A pre-irradiation kV-CBCT image was obtained using the image acquisition and reconstruction parameters in Table 2. Subsequently, the gel dosimeter was irradiated with seven narrow beams using gantry and couch rotations (Fig. 1B), and post-irradiated kV-CBCT images were acquired using the same parameters as above (Fig. 1C). For analysis, the image datasets of pre-irradiated and post-irradiated gel dosimeters were imported into our in-house program^25^. The analysis quantified the minimum distance ($\:{d}_{\text{m}\text{i}\text{n}}$) between each radiation beam and the origin of the imaging coordinate system (imaging isocenter, $\:\text{i}\text{I}\text{C}$), the distance ($\:{d}_{\text{i}\text{I}\text{C}-\text{r}\text{I}\text{C}}$) between the radiation isocenter ($\:\text{r}\text{I}\text{C}$) and the $\:\text{i}\text{I}\text{C}$, and the radius ($\:r$) of the smallest sphere that intersects all beams^25^.

**Table 1:** Gantry, couch, and collimator angles of beams used in this study, following International Electrotechnical Commission standard 61217.

Beam No.	Gantry [degree]	Couch [degree]	Collimator [degree]
1	45	0	0
2	135	0	0
3	180	0	0
4	270	0	0
5	45	45	0
6	45	90	0
7	45	270	0

**Table 2:** Kilovoltage cone-beam CT image acquisition and reconstruction parameters used for the three-dimensional starshot analysis.

Parameter	Varian (iCBCT)	Varian (FBP)	Elekta
Tube voltage [kV]	80	80	70
Tube current-exposure time product [mAs]	1080	1080	2112
Frame rate [frames/s]	15	15	5.5
Gantry speed [degree/s]	1	1	0.6
Rotational angle [degree - degree]	180–180 (CCW)	180–180 (CCW)	180–180 (CCW)
Fan type	Full	Full	Full
Trajectory	Full	Full	Full
Reconstruction	iCBCT	FBP	Feldkamp
Matrix size	512 × 512	512 × 512	512 × 512
Pixel size [mm^2^]	0.51 × 0.51	0.51 × 0.51	0.51 × 0.51
Slice thickness[mm]	1	1	1
The other reconstruction parameters	Smooth reconstruction filter: standard denoise: Medium	Smooth reconstruction filter: Auto	Reconstruction filter: Wiener Interpolation: strong (set as Partial2) Scatter Correction: Uniform (set the factor as 0.24) Pre Filter: Median (strong) Projection Down Size Factor: 1 (best quality)

*iCBCT* iterative cone-beam computed tomography, *FBP* filtered back projection, *CCW* counterclockwise.

The values of $\:{d}_{\text{m}\text{i}\text{n}}$, $\:{d}_{\text{i}\text{I}\text{C}-\text{r}\text{I}\text{C}}$, and $\:r$ were obtained at all institutions. An inter-manufacturer comparison was made for these values. For $\:{d}_{\text{m}\text{i}\text{n}}$ and $\:{d}_{\text{i}\text{I}\text{C}-\text{r}\text{I}\text{C}}$, the comparison was made for each direction of the image (DICOM) reference coordinate system and for the resultant vector.

### Impact of 3D linac accuracy on SRS

We assessed the impact of the spatial dose delivery accuracy on the PTV margins in intracranial SRS. PTV margins were derived using the 3D-SS analysis results. Zhang et al. proposed a method to calculate the optimal PTV margin for single-fraction intracranial SRS that incorporates the linac spatial dose delivery error and the residual patient setup error after image guidance^19,20^. The method provides an anisotropic margin for intracranial SRS that ensures with 95% probability that the clinical target volume receives the prescribed dose.

In this study, we developed the method by inputting the 3D dose delivery accuracy derived from the 3D-SS test as the linac spatial dose delivery error, building on a previously reported study^20^. After calculating the optimal PTV margins, inter-institution and inter-manufacturer comparisons were made.

The formulas are

1 $$\:{C}_{i}={W}_{0i}+{b}_{1}\left({W}_{0i}\right){\sigma\:}_{i}+{b}_{2}\left({W}_{0i}\right){\sigma\:}_{i}^{2},$$

2 $${b}_{1}\left({W}_{0i}\right)={2.331-1.425}W_{0i}+2.296{W}_{0i}^{2}-1.539{W}_{0i}^{3}+0.374{W}_{0i}^{4},$$

3 $${b}_{2}\left({W}_{0i}\right)={0.434W}_{0i}-0.917{W}_{0i}^{2}+0.676{W}_{0i}^{3}-0.171{W}_{0i}^{4},$$

where $\:{C}_{i}$ represents the PTV margin for the *i* = *X*, *Y*, and *Z* directions in the DICOM reference coordinate system, which is correspond to the patient right-left, patient superior-inferior, and patient anterior-posterior directions, respectively; $\:{W}_{0i}={V}_{s0i}+{V}_{r0i}$, where $\:{V}_{s0i}$ represents the systematic error in the imaging isocenter and radiation isocenter along each axis; $\:{V}_{r0i}$ denotes the average residual error in patient setup after image guidance; and $\:{\sigma\:}_{i}$ is a function of the standard deviation of the residual error in patient setup ($\:{SD}_{\text{s}\text{e}\text{t}\text{u}\text{p},i}$). In this study, we added fluctuations in the radiation beam position (random error in the radiation isocenter) to $\:{\sigma\:}_{i}$:

4 $$\:{\sigma\:}_{i}=\sqrt{{SD}_{\text{s}\text{e}\text{t}\text{u}\text{p},i}^{2}+{\left(0.683\times\:r\right)}^{2}},$$

where the term $\:0.683\times\:r$ corresponds to the standard deviation of the radiation isocenter error, which was assumed to follow a normal distribution.

In this study, $\:{V}_{s0i}$ was directly set equal to $\:{d}_{\text{i}\text{I}\text{C}-\text{r}\text{I}\text{C}}$, which includes the influence of couch rotation. The values of $\:{V}_{r0i}$ and $\:{SD}_{\text{s}\text{e}\text{t}\text{u}\text{p},i}$ were taken from the studies of Zhang et al.^20^ and Ong et al.^26^ and are shown in Supplementary Table A1. The values of the former and the latter were defined as the larger and smaller residual patient setup errors.

### Impact of QA procedures on SRS accuracy

We evaluated the impact of implementing advanced image guidance systems for patient positioning and rigorous geometric QA of the linac. This study assumed that advanced image guidance systems, which include widely installed video-based 3D optical surface imaging and 2D kV stereoscopic imaging, were implemented. During non-coplanar irradiation in clinical settings, we also assumed that intra-fraction patient motion caused by treatment couch shifts during couch rotation could be monitored and corrected by these imaging modalities. For example, we hypothesized that current surface-guided radiation therapy systems could detect a couch walk-out of < 1 mm^31^. Furthermore, the value of $\:{d}_{\text{i}\text{I}\text{C}-\text{r}\text{I}\text{C}}$ was treated as a systematic error for linacs that could be minimized by a geometric calibration test performed by the user. These assumptions form the basis for the further analysis conducted in this study. Therefore, this study assumed that in clinical practice, the user’s efforts can reduce the impact of these two factors on geometric accuracy to clinically negligible values. Based on this assumption, the PTV margin was recalculated by replicating the situation using the following procedure: 3D dose delivery accuracy was recalculated using only the co-planar beams, and the new values of $\:{d}_{\text{i}\text{I}\text{C}-\text{r}\text{I}\text{C}}$ and $\:r$ were measured. Subsequently, $\:{d}_{\text{i}\text{I}\text{C}-\text{r}\text{I}\text{C}}$ was set to zero to account for the error contribution of the geometric calibration of the linac. Finally, the PTV margin was recalculated using Eqs. 1–4 and the above value of $\:r$.

### Statistical analysis

Statistical analyses were performed to examine inter-manufacturer differences in various parameters. The Kruskal–Wallis test and Mann–Whitney *U* test were used to compare the results from more than two institutions and vendors, respectively. The Steel–Dwass post hoc test was used to make pair-wise comparisons. All statistical analyses were conducted using EZR (version 1.62, Saitama Medical Center, Jichi Medical University, Saitama, Japan). A value of *p* < 0.05 was considered statistically significant.

## Results

**Fig. 2:**
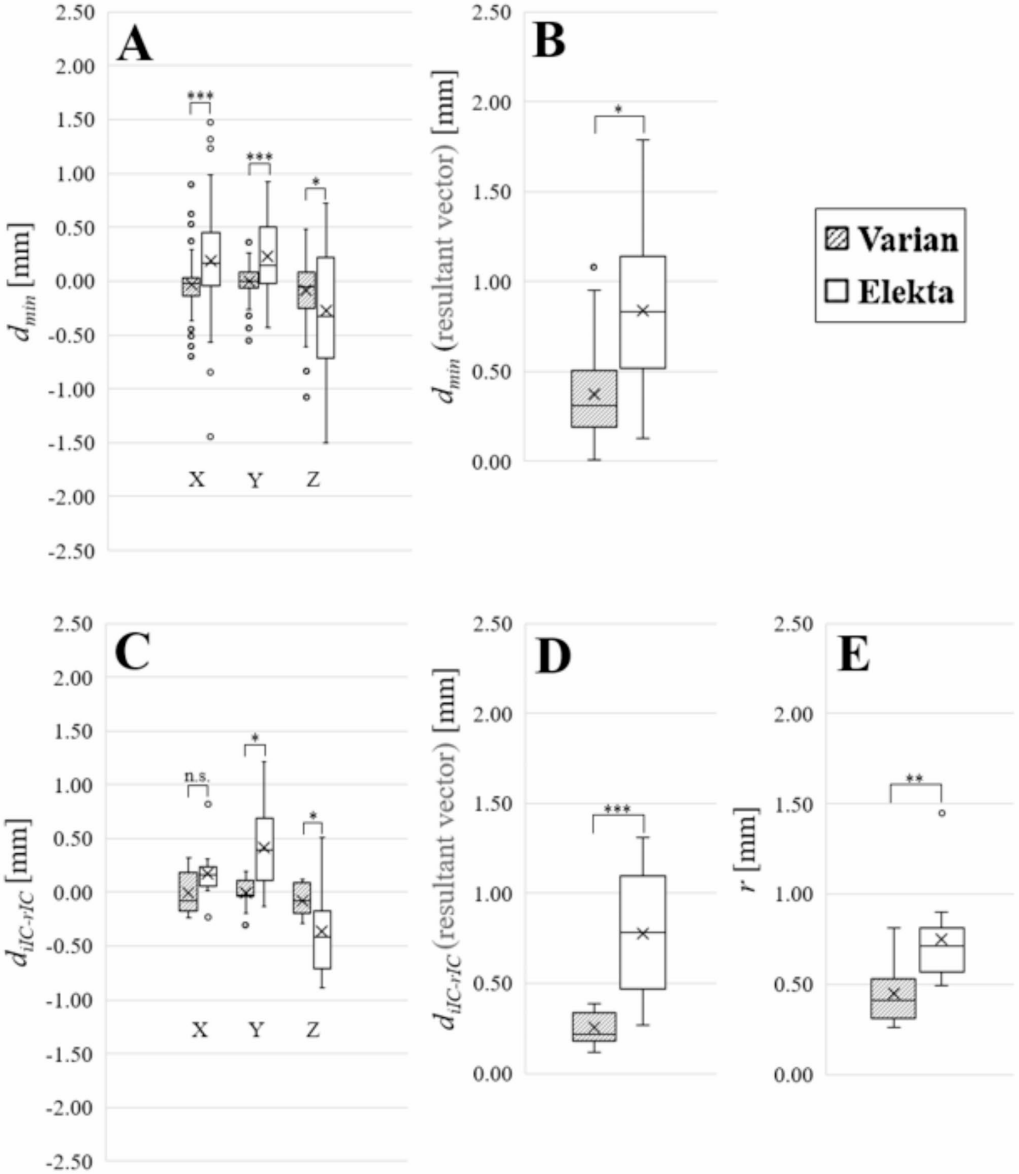
Box and whisker plots of the 3D-SS test performed using Varian and Elekta machines at 22 institutions (bold line, median; cross, mean; boxes, first and third quartiles; whiskers, 5th and 95th percentiles; circles, outliers). (A) Minimum distance ($\:{d}_{\text{m}\text{i}\text{n}}$) of the seven beams from the imaging isocenter in three directions. (B) Value of $\:{d}_{\text{m}\text{i}\text{n}}$ for the seven beams, expressed in terms of the resultant vector. (C) Distance between the imaging isocenter and radiation isocenter ($\:{d}_{\text{i}\text{I}\text{C}-\text{r}\text{I}\text{C}}$) in three directions. (D) Value of $\:{d}_{\text{i}\text{I}\text{C}-\text{r}\text{I}\text{C}}$ expressed in terms of the resultant vector. (E) Radius ($\:r$) of the smallest sphere that intersects all radiation beams. Notation: n.s. = not significant; *p < 0.05; **p < 0.01; ***p < 0.001. The positive and negative symbols on $\:{d}_{\text{m}\text{i}\text{n}}$ and $\:{d}_{\text{i}\text{I}\text{C}-\text{r}\text{I}\text{C}}$ indicate directions in DICOM reference coordinate systems.

Figure 2 categorizes the values of $\:{d}_{\text{m}\text{i}\text{n}}$, $\:{d}_{\text{i}\text{I}\text{C}-\text{r}\text{I}\text{C}}$, and $\:r$ for non-coplanar irradiation for each institution according to the linac manufacturer, and the raw data for these parameters are summarized in Supplements B, C, and D, respectively.

For the seven beams used at each institution, $\:{d}_{\text{m}\text{i}\text{n}}\:$showed variability among institutions and between manufacturers (Fig. 2A and Supplements B1, B2). As shown in Fig. 2A, the positional errors in the beam paths of the Elekta systems were significantly larger than those of the Varian systems in the *X* ($\:p\:$< 0.001), *Y* ($\:p$ < 0.001), and *Z* ($\:p$ < 0.05) directions. In terms of the resultant vector, the Elekta systems had a significantly larger positional error in the beam path than the Varian systems $\:(p\:$< 0.05), as shown in Fig. 2B, and the numbers of institutions for which the value exceeded 1 mm were one and nine for Varian and Elekta systems, respectively. These 10 institutions exceeded the 1 mm tolerance proposed for the E2E test by the Medical Physics Practice Guidelines developed by the American Association of Physicists in Medicine and also proposed in the consensus statement from the working groups for radiosurgery and stereotactic radiotherapy of the German Society for Radiation Oncology and for physics and technology in stereotactic radiotherapy of the German Society for Medical Physics^13,15^.

The value of $\:{d}_{\text{i}\text{I}\text{C}-\text{r}\text{I}\text{C}}$, which includes the influence of couch rotation, was evaluated. As shown in Fig. 2C and Supplement C, the values of $\:{d}_{\text{i}\text{I}\text{C}-\text{r}\text{I}\text{C}}$ for the Varian systems were within ± 0.32 mm in all directions, and the median values were within 0.1 mm. In contrast, the Elekta systems had a maximum $\:{d}_{\text{i}\text{I}\text{C}-\text{r}\text{I}\text{C}}$ of 1.21 mm in the *Y* direction, with median values in the *X*, *Y*, and *Z* directions of 0.16, 0.39, and − 0.42 mm, respectively. The displacements for the Elekta systems were significantly larger than those for the Varian systems ($\:p$ < 0.001), as shown in Fig. 2D and Supplement C.

The radius of the smallest sphere intersecting all radiation beams, which includes the effect of couch rotation, is presented in Fig. 2E and Supplement D. The Elekta linacs had a significantly larger radius ($\:p\:$< 0.001) than the Varian linacs.

The correlation coefficients were derived from a linear regression analysis of the relationship between $\:{d}_{\text{i}\text{I}\text{C}-\text{r}\text{I}\text{C}}$ and the number of operational years of each machine, as well as between $\:r$ and the number of operational years of each machine. As shown in Table 3, Varian linacs had no correlation in terms of increments in $\:{d}_{\text{i}\text{I}\text{C}-\text{r}\text{I}\text{C}}$ and $\:r$ with the number of operational years of each machine. However, moderate correlations were observed for Elekta, indicating that $\:{d}_{\text{i}\text{I}\text{C}-\text{r}\text{I}\text{C}}$ and $\:r$ increased as the machine life advanced.

**Table 3:** Coefficients of correlation of $\:{d}_{\text{i}\text{I}\text{C}-\text{r}\text{I}\text{C}}$ and $\:r$ with the number of operational years of each machine

	Varian	Elekta
$\:{d}_{\text{i}\text{I}\text{C}-\text{r}\text{I}\text{C}}$	0.13	0.52
$\:r$	-0.52	0.59

**Fig. 3:**
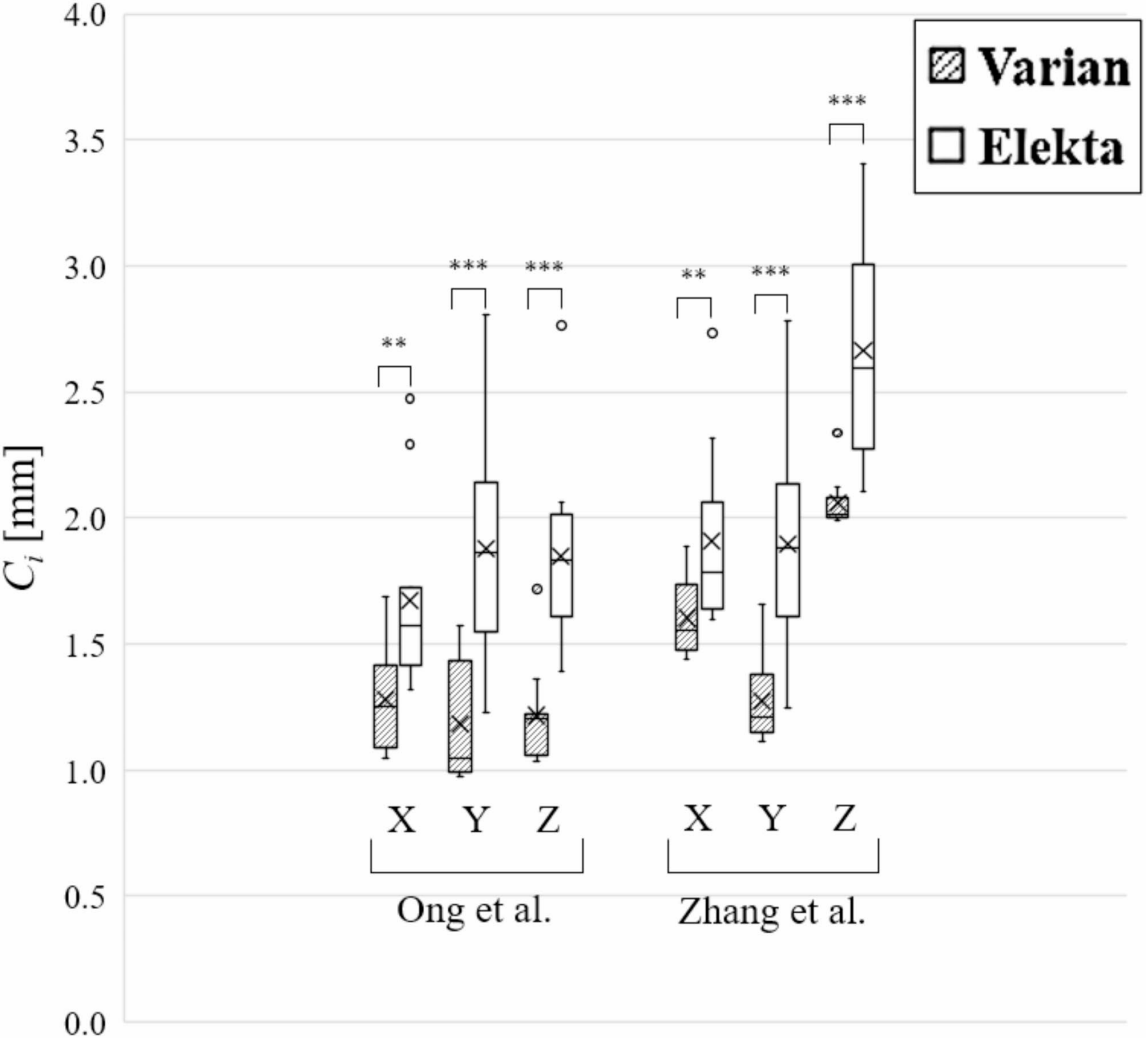
Box and whisker plots of the planning target volume (PTV) margins ($\:{C}_{i}$; *i* = *X*, *Y*, *Z*) for intracranial single-fraction stereotactic radiosurgery at 22 institutions. The margin was derived from 3D-SS analysis of the dose delivery accuracy and from residual setup errors reported by Ong et al.^26^ and Zhang et al.^20^. Notation: *p < 0.05; **p < 0.01; ***p < 0.001.

**Fig. 4:**
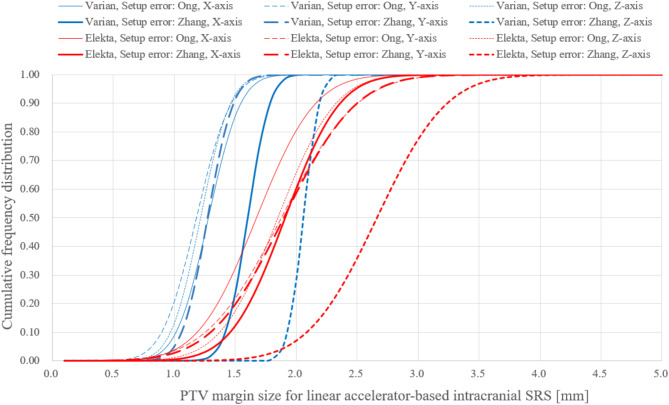
Cumulative frequency distributions of the PTV margins for Varian and Elekta systems used in this study. The margin sizes in three directions are shown, and they were derived from 3D-SS analysis of the dose delivery accuracy and from residual setup errors reported by Ong et al.^26^ and Zhang et al.^20^.

Figure 3 and Supplement E show the PTV margins derived from 3D-SS analysis of dose delivery error and from residual patient setup errors after image guidance. The PTV margins varied among institutions and between manufacturers. The minimum and maximum PTV margins were 1.0 and 3.4 mm, respectively, regardless of manufacturer or coordinate axis. For Varian, the margin ranges under the assumed smaller and larger setup errors were 1.0–1.9 mm, 1.0–1.7 mm, and 1.0–2.3 mm in the X, Y, and Z directions, respectively. For Elekta, the margin ranges under the two conditions were 1.3–2.7 mm, 1.2–2.8 mm, and 1.4–3.4 mm in the X, Y, and Z directions, respectively. Significant differences in the PTV margins between the Varian and Elekta systems were measured in the *X* ($\:p$ < 0.01), *Y* ($\:p$ < 0.01), and *Z* ($\:p$ < 0.001) directions. Assuming that the PTV margins calculated for the institutions sampled follow a normal distribution, the cumulative frequency distributions of the PTV margins were calculated for each manufacturer (Fig. 4). The probability of exceeding a 2 mm margin was higher for Elekta systems than for Varian systems. Table 4 shows the PTV margins that could cover the GTV in the *X*, *Y*, and *Z* directions with 95% probability at 95% of the institutions. A PTV margin of 2 mm was achievable for the Varian systems if the smaller setup errors reported by Ong et al.^26^ were assumed. The margin for Elekta systems was unachievable even under the assumed smaller and larger setup errors.

**Table 4:** Planning target volume (PTV) margins for Varian and Elekta linear accelerators that could cover the gross tumor volume with 95% probability at 95% of the institutions.

Manufacturer	Direction	PTV margin size [mm]	
		Ong	Zhang
Varian	X	1.7	1.9
	Y	1.6	1.6
	Z	1.6	2.3
Elekta	X	2.4	2.6
	Y	2.8	2.8
	Z	2.6	3.5

The margins in three directions are shown, and they were derived from a three-dimensional starshot (3D-SS) test of the dose delivery accuracy and the residual setup errors reported by Ong et al.^26^ and Zhang et al.^20^.

Assuming that high-accuracy 3D dose delivery, strict patient immobilization and intra-fractional positioning, and rigorous linac QA could improve 3D geometric accuracy, the isocentricity $\:r$ was reduced from 0.81 to 0.32 mm for Varian systems and from 1.45 to 0.76 mm for Elekta systems (cf. Supplements D and F). Being less than 1 mm, these values met the proposed tolerance level^13,15^. PTV margins for all institutions are shown in Table 5, and those margins that could cover the GTV with 95% probability at 95% of the institutions are shown in Table 6. Even when a larger residual patient setup error was assumed, the margin size for all institutions using Varian systems remained below 2 mm, and the anisotropic PTV margins that could cover the GTV with 95% probability at 95% of the institutions were 1.5, 1.1, and 2.0 mm in the *X*, *Y*, and *Z* directions, respectively. When a smaller residual patient setup error was assumed, the PTV margin size for all institutions using Elekta systems remained below 2 mm, and the isotropic margins that could cover the GTV with 95% probability at 95% of the institutions were 1 mm for Varian systems and 1.5 mm for Elekta systems.

**Table 5:** PTV margin for each institution, determined assuming that $\:{d}_{\text{i}\text{I}\text{C}-\text{r}\text{I}\text{C}}$ is zero owing to implementation of rigorous QA procedures and that intra-fraction error caused by treatment couch rotation is zero owing to implementation of a real-time positional monitoring system.

Setup error (reference)			Ong et al.			Zhang et al.		
Manufacturer	Institution	Years of operation	X	Y	Z	X	Y	Z
Varian	A	10	1.0	0.9	0.9	1.4	1.1	1.9
	B	7	1.0	0.9	1.0	1.4	1.1	2.0
	C	7	1.0	0.9	1.0	1.4	1.1	2.0
	D	5	1.0	0.9	1.0	1.4	1.1	2.0
	E	5	1.0	0.9	0.9	1.4	1.1	1.9
	F	6	1.0	0.9	1.0	1.4	1.1	2.0
	G	7	1.0	0.9	1.0	1.4	1.1	2.0
	H	9	1.0	0.9	1.0	1.5	1.1	2.0
	I	7	1.0	0.9	1.0	1.4	1.1	2.0
	J	6	1.0	0.9	0.9	1.4	1.1	1.9
	K	1	1.0	0.9	1.0	1.4	1.1	2.0
	Minimum		1.0	0.9	0.9	1.4	1.1	1.9
	Maximum		1.0	0.9	1.0	1.5	1.1	2.0
	Average		1.0	0.9	1.0	1.4	1.1	2.0
	Standard deviation		0.0	0.0	0.0	0.0	0.0	0.0
Elekta	L	6	1.3	1.3	1.3	1.7	1.4	2.2
	M	5	1.5	1.5	1.5	1.8	1.6	2.3
	N	5	1.2	1.1	1.2	1.6	1.3	2.1
	O	12	1.3	1.3	1.3	1.7	1.4	2.1
	P	7	1.3	1.2	1.3	1.6	1.4	2.1
	Q	12	1.3	1.3	1.3	1.7	1.4	2.2
	R	11	1.4	1.3	1.3	1.7	1.4	2.2
	S	4	1.1	1.0	1.0	1.5	1.2	2.0
	T	9	1.3	1.3	1.3	1.7	1.4	2.1
	U	7	1.3	1.3	1.3	1.7	1.4	2.1
	V	0	1.2	1.1	1.2	1.6	1.3	2.1
	Minimum		1.1	1.0	1.0	1.5	1.2	2.0
	Maximum		1.5	1.5	1.5	1.8	1.6	2.3
	Average		1.3	1.2	1.3	1.7	1.4	2.1
	Standard deviation		0.1	0.1	0.1	0.1	0.1	0.1

Intra-fraction setup errors reported by Ong et al.^26^ and Zhang et al.^20^ have also been applied.

**Table 6:** PTV margins for Varian and Elekta linear accelerators that could cover the gross tumor volume with 95% probability at 95% of the institutions, determined assuming that the distance between the imaging and radiation isocenters is zero owing to rigorous QA implementation and that the intra-fraction error caused by treatment couch rotation is zero owing to implementation of a positional monitoring system such as surface image guidance.

Manufacturer	Direction	Reduced PTV margin size [mm]	
		Ong	Zhang
Varian	X	1.0	1.5
	Y	1.0	1.1
	Z	1.0	2.0
Elekta	X	1.5	1.9
	Y	1.5	1.6
	Z	1.5	2.3

The margins in three directions are shown, and they were derived from 3D-SS analysis of the dose delivery accuracy and the residual setup errors reported by Ong et al.^26^ and Zhang et al.^20^.

## Discussion

To our knowledge, this is the first multi-institutional study to combine 3D-SS analysis with an X-ray CT-based polymer gel dosimeter to evaluate the 3D dose delivery accuracy, including couch rotation accuracy, of commercially available linacs broadly in clinical use. Moreover, institution- and manufacturer-specific PTV margins for intracranial SRS were derived from 3D-SS analysis of dose delivery accuracy. This report will serve as a useful reference for institutions seeking to objectively evaluate their own dose delivery accuracy and to determine the PTV margin for intracranial SRS.

This study made several important findings. First, it provides generally achievable values for linacs from each manufacturer. Pant et al. proposed a 3D-SS method^23^, and Oshika et al. demonstrated that it can minimize measurement uncertainties^25^. However, there is no information on variations in 3D dose delivery accuracy among commercially available linacs.

Second, we found that one Varian and nine Elekta linacs exceeded the 1 mm tolerance of SRS guidelines on spatial irradiation accuracy, even if conventional WL tests and timings were followed. In a previous study, there were discrepancies of < 0.4 mm in the resulting coincidence of each beam obtained via the conventional WL test and 3D-SS test^25^. Thus, these results imply that conventional QA methods may underestimate 3D dose delivery errors. A 3D QA procedure with low measurement uncertainties, such as the 3D-SS test, should be performed to achieve dose delivery errors of no more than 1 mm in 3D coordinates.

Third, non-coplanar irradiation for intracranial SRS performed without any image guidance system may need more than a 2 mm PTV margin when a larger residual patient setup error is assumed. Several studies have reported that the PTV margin can be reduced without affecting the clinical outcome^9,11^. However, it is unclear whether similar results can be obtained at other institutions because our findings indicate that the accuracy of irradiation is institution-dependent. In this study, the margin was in the 1.0–3.4 mm range. This considerable variation indicates that universal determination and reduction of the PTV margin should be approached cautiously.

Fourth, older machines demonstrated lower precision than newer machines in only Elekta. Varian machines did not show the trends in this study. Gao J et al. compared the number of years of operation and the results of the off-iso Winston–Lutz test for Varian machines, and found that newer models had higher accuracy than older models^32^. Because all the machines in this study were of the same model—TrueBeam and TrueBeam STx—it is likely that this resulted in differing outcomes.

Collimator rotation was not considered in the 3D-SS test for this study. The 3D dose delivery accuracy and PTV margin may, therefore, be greater than the values reported in this study. Because a single gel dosimeter was used for the 3D-SS test and all beam trajectories were included within the dosimeter, the number of beams tested was limited. To assess the impact of collimator rotation, non-coplanar beams with varying collimator angles could be used in a separate gel dosimeter test. In addition, the margin formula established in this study does not account for factors such as inter-observer variations in target volume delineation among physicians or registration errors in planning CT and MRI. Therefore, considering these factors, the required margin may potentially increase further.

In this study, we used linacs for intracranial stereotactic radiotherapy in clinical practice. Although the Elekta linac models used in this study belong to different generations, there are no differences in the structure and function of the linacs across the three models. The couch systems differ among the three models. However, only the yaw rotation of the couch was used in this study, and we considered that there was no significant difference in the accuracy of the yaw rotation among the models.

It is important to note that the single-isocenter, multi-target (SIMT) delivery technique has gained increasing popularity. However, its heightened sensitivity to rotational errors in dose delivery to small off-isocenter targets presents significant challenges for margin calculation using analytical methods (e.g., the margin depends on the specific distance of each target from the isocenter). This underscores the critical advantage of 3D dose measurement, which provides superior accuracy and reliability compared to conventional methods.

Finally, this study highlights the importance of precise management of the 3D dose delivery accuracy by medical physicists. Radiation oncology departments should also introduce a real-time monitoring system capable of correcting patient displacement due to body movement and couch rotation. Moreover, therapists must improve patient immobilization and verification of position accuracy. The margin could be reduced to within the recommended 2 mm limit by assuring high-accuracy 3D dose delivery, strict patient immobilization and intra-fractional positioning, and rigorous linac QA^12^. Our multi-institutional study showed that it is possible to limit the PTV margin size for intracranial SRS to 1.0 mm for Varian systems and 1.5 mm for Elekta systems in all directions.

## Conclusion

The 3D dose delivery accuracy of linacs currently in operation largely varied at the millimeter level in this study. The accuracy of current radiotherapy technology should not be overestimated, and it is essential to rigorously determine the 3D dose delivery accuracy and estimate the PTV margins. Using advanced image guidance systems with limited patient setup error and recognizing the accuracy variations between linear accelerators, it is essential to maximize a linac’s 3D dose delivery accuracy to achieve the required PTV margin in intracranial SRS.

## Electronic supplementary material

Below is the link to the electronic supplementary material.

Supplementary Material A

Supplementary Material B

Supplementary Material C

Supplementary Material D

Supplementary Material E

Supplementary Material F

## Data Availability

Research data are stored in an institutional repository and will be shared upon request to the corresponding author.
