# Supplementary material for: Spatial accuracy of dose delivery significantly impacts the planning target volume margin in linear accelerator-based intracranial stereotactic radiosurgery
Source: Sci Rep. 2025 Jan 29;15:3608. doi: 10.1038/s41598-025-87769-z (PMC11775166; doi:10.1038/s41598-025-87769-z)
Supplement: Supplementary file 1 — Supplementary Material A [file 41598_2025_87769_MOESM1_ESM.pdf]

Supplementary Table A: Intra-fraction errors reported by Ong et al.,[26] and residual setup errors reported by Zhang et al.[20]

	Ong et al. (averaged value of two institutions)		Zhang et al.	
	Mean	Standard deviation	Mean	Standard deviation
Lateral (X)	0.14	0.35	-0.17	0.59
Vertical (Y)	0.23	0.26	-0.03	0.43
Longitudinal (Z)	0.03	0.37	0.10	0.81
