# Supplementary material for: Spatial accuracy of dose delivery significantly impacts the planning target volume margin in linear accelerator-based intracranial stereotactic radiosurgery
Source: Sci Rep. 2025 Jan 29;15:3608. doi: 10.1038/s41598-025-87769-z (PMC11775166; doi:10.1038/s41598-025-87769-z)
Supplement: Supplementary file 2 — Supplementary Material B [file 41598_2025_87769_MOESM2_ESM.pdf]

Supplement B1: Minimum distance ($d_{\min}$) between the imaging isocenter and each beam, determined at institutions with systems manufactured by Varian Medical Systems. The positive and negative symbols on $d_{\min}$ indicate directions in DICOM reference coordinate systems.

Manufacture	Institution	Linear accelerator	Years of operation	Beam No.	Gantry angle [degree]	Couch angle [degree]	$d_{\min}$ [mm]			
							X	Y	Z	Vector
Varian	A	TrueBeam	10	1	45	0	0.13	0.13	-0.07	0.20
				2	135	0	-0.16	0.16	-0.44	0.50
				3	180	0	-0.04	0.00	-0.45	0.45
				4	270	0	0.00	0.03	-0.43	0.43
				5	45	45	-0.13	0.05	-0.20	0.25
				6	45	90	-0.06	-0.07	0.07	0.11
				7	45	270	0.62	0.15	0.15	0.66
	B	TrueBeamSTx	7	1	45	0	-0.03	-0.03	-0.09	0.10
				2	135	0	-0.05	0.05	-0.50	0.50
				3	180	0	-0.04	0.00	-0.61	0.61
				4	270	0	0.00	0.16	-0.31	0.35
				5	45	45	-0.37	-0.10	-0.23	0.45
				6	45	90	-0.51	0.08	-0.08	0.52
				7	45	270	0.16	-0.06	-0.06	0.18
	C	TrueBeamSTx	7	1	45	0	0.02	0.02	0.39	0.39
				2	135	0	-0.09	0.08	-0.10	0.15
				3	180	0	-0.10	0.00	-0.14	0.17
				4	270	0	0.00	0.01	0.00	0.01
				5	45	45	0.02	-0.08	0.15	0.17
				6	45	90	-0.09	-0.02	0.02	0.10
				7	45	270	0.14	0.06	0.07	0.17
	D	TrueBeam	5	1	45	0	0.07	0.07	0.04	0.10
				2	135	0	-0.02	0.02	-0.28	0.28
				3	180	0	-0.06	0.00	-0.48	0.48
				4	270	0	0.00	0.04	-0.27	0.27
				5	45	45	-0.28	-0.10	-0.14	0.33
				6	45	90	-0.29	0.11	-0.11	0.33
				7	45	270	0.53	-0.10	-0.10	0.55
	E	TrueBeam	5	1	45	0	0.10	0.10	0.48	0.50
				2	135	0	0.03	-0.03	0.15	0.16

			3	180	0	-0.06	0.00	0.08	0.10
			4	270	0	0.00	0.19	0.16	0.25
			5	45	45	0.12	0.00	0.12	0.17
			6	45	90	0.41	0.22	-0.22	0.51
			7	45	270	-0.56	-0.04	-0.05	0.57
F	TrueBeam	6	1	45	0	-0.03	-0.03	0.29	0.29
			2	135	0	0.12	-0.12	-0.23	0.28
			3	180	0	0.00	0.00	-0.28	0.28
			4	270	0	0.00	0.36	-0.04	0.36
			5	45	45	-0.14	-0.07	-0.04	0.16
			6	45	90	-0.01	0.02	-0.02	0.02
			7	45	270	-0.29	-0.32	-0.32	0.54
G	TrueBeamSTx	7	1	45	0	-0.06	-0.06	0.39	0.40
			2	135	0	-0.17	0.17	0.00	0.24
			3	180	0	-0.08	0.00	-0.22	0.23
			4	270	0	0.00	0.08	0.08	0.11
			5	45	45	-0.20	-0.25	0.15	0.35
			6	45	90	-0.23	-0.15	0.15	0.31
			7	45	270	0.14	0.13	0.13	0.23
H	TrueBeamSTx	9	1	45	0	-0.45	-0.44	0.04	0.63
			2	135	0	-0.02	0.02	-0.29	0.29
			3	180	0	-0.29	0.00	-0.44	0.52
			4	270	0	0.00	0.21	-0.15	0.26
			5	45	45	-0.70	-0.55	0.09	0.90
			6	45	90	-0.47	-0.39	0.40	0.73
			7	45	270	0.29	-0.14	-0.14	0.35
I	TrueBeam	7	1	45	0	0.00	0.00	0.48	0.48
			2	135	0	-0.15	0.15	0.02	0.21
			3	180	0	-0.14	0.00	-0.10	0.17
			4	270	0	0.00	0.05	0.07	0.09
			5	45	45	0.37	0.05	0.30	0.48
			6	45	90	0.90	0.10	-0.11	0.91
			7	45	270	-0.28	-0.26	-0.27	0.47
J	TrueBeam	6	1	45	0	-0.03	-0.03	0.41	0.41
			2	135	0	0.11	-0.11	0.13	0.20
			3	180	0	0.04	0.00	0.07	0.08
			4	270	0	0.00	0.26	0.06	0.27

			5	45	45	0.03	-0.14	0.23	0.27
			6	45	90	0.23	-0.03	0.03	0.23
			7	45	270	-0.10	-0.18	-0.19	0.28
			1	45	0	0.11	0.11	-0.51	0.53
			2	135	0	-0.08	0.08	-1.07	1.08
			3	180	0	-0.02	0.00	-1.08	1.08
K	TrueBeam	1	4	270	0	0.00	0.05	-0.84	0.84
			5	45	45	-0.43	-0.02	-0.41	0.59
			6	45	90	-0.60	0.01	-0.01	0.60
			7	45	270	0.95	0.06	0.06	0.95
					Minimum	-0.70	-0.55	-1.08	0.01
					Maximum	0.95	0.36	0.48	1.08
					Median	-0.02	0.00	-0.04	0.31
					Average	-0.03	-0.01	-0.08	0.38
					Standard deviation	0.31	0.16	0.32	0.26

Supplement B2 Minimum distance (d_{min}) between the imaging isocenter and each beam, determined at institutions with systems manufactured by Elekta AB. The positive and negative symbols on d_{min} indicate directions in DICOM reference coordinate systems.

Manufacture	Institution	Linear accelerator	Years of operation	Beam No.	Gantry angle [degree]	Couch angle [degree]	d_{min} [mm]			
							X	Y	Z	Vector
Elekta	L	VersaHD	6	1	45	0	0.26	0.26	0.30	0.47
				2	135	0	-0.57	0.57	-0.65	1.03
				3	180	0	-0.85	0.00	-0.78	1.15
				4	270	0	0.00	-0.06	-0.20	0.20
				5	45	45	0.62	0.46	-0.03	0.77
				6	45	90	0.35	0.72	-0.73	1.08
				7	45	270	-0.41	0.49	0.50	0.81
	M	VersaHD	5	1	45	0	-0.34	-0.34	0.20	0.53
				2	135	0	0.26	-0.26	-0.88	0.95
				3	180	0	0.50	0.00	-0.88	1.01
				4	270	0	0.00	0.86	-0.42	0.96
				5	45	45	0.17	-0.20	0.47	0.54
				6	45	90	0.41	-0.31	0.32	0.61
				7	45	270	0.82	-0.35	-0.35	0.95
	N	Infinity	5	1	45	0	0.46	0.46	-0.30	0.72
				2	135	0	-0.11	0.11	-1.21	1.22
				3	180	0	0.09	0.00	-1.32	1.32
				4	270	0	0.00	0.85	-0.71	1.10
				5	45	45	0.56	0.74	-0.50	1.05
				6	45	90	0.29	0.92	-0.93	1.34
				7	45	270	-0.12	-0.03	-0.03	0.13
	O	Synergy	12	1	45	0	0.69	0.69	0.38	1.05
				2	135	0	0.00	0.00	-0.42	0.42
				3	180	0	0.26	0.00	-0.64	0.69
				4	270	0	0.00	-0.35	-0.08	0.36
				5	45	45	1.26	0.82	0.10	1.51
				6	45	90	1.32	0.84	-0.86	1.79
				7	45	270	0.04	0.12	0.12	0.18
	P	Infinity	7	1	45	0	0.51	0.51	-0.21	0.75
				2	135	0	-0.49	0.50	-1.17	1.37

			3	180	0	-0.20	0.00	-1.16	1.17
			4	270	0	0.00	0.01	-0.66	0.66
			5	45	45	0.57	0.80	-0.58	1.14
			6	45	90	0.33	0.84	-0.86	1.25
			7	45	270	-0.07	0.26	0.27	0.38
			1	45	0	0.80	0.80	0.69	1.32
			2	135	0	-0.55	0.55	0.44	0.89
			3	180	0	-0.80	0.00	0.23	0.83
Q	Synergy	12	4	270	0	0.01	0.33	0.57	0.66
			5	45	45	1.23	0.43	0.61	1.44
			6	45	90	0.94	0.43	-0.45	1.13
			7	45	270	-0.33	0.58	0.61	0.90
			1	45	0	0.22	0.22	0.72	0.78
			2	135	0	-0.02	0.02	-0.38	0.38
			3	180	0	0.27	0.00	-0.55	0.62
R	Synergy	11	4	270	0	0.00	0.28	0.01	0.28
			5	45	45	0.99	0.46	0.35	1.14
			6	45	90	1.47	0.40	-0.41	1.58
			7	45	270	-1.44	-0.16	-0.19	1.46
			1	45	0	-0.32	-0.30	-0.49	0.66
			2	135	0	-0.41	0.42	-0.97	1.14
			3	180	0	-0.27	-0.01	-0.96	1.00
S	VersaHD	4	4	270	0	0.00	-0.43	-0.52	0.67
			5	45	45	0.67	0.71	-0.33	1.03
			6	45	90	0.66	0.73	-0.73	1.23
			7	45	270	-0.20	-0.24	-0.24	0.39
			1	45	0	0.42	0.42	-0.28	0.65
			2	135	0	0.16	-0.16	-1.12	1.15
			3	180	0	0.00	0.00	-1.50	1.50
T	Infinity	9	4	270	0	0.01	0.15	-0.71	0.73
			5	45	45	0.43	0.60	-0.42	0.85
			6	45	90	0.08	0.81	-0.82	1.15
			7	45	270	0.29	0.23	0.24	0.44
			1	45	0	-0.18	-0.19	0.72	0.76
U	Synergy	7	2	135	0	0.23	-0.24	-0.20	0.39
			3	180	0	0.35	0.00	-0.36	0.50
			4	270	0	0.00	0.47	0.30	0.56

			5	45	45	0.66	0.03	0.64	0.92
			6	45	90	0.76	-0.04	0.05	0.77
			7	45	270	-0.25	-0.14	-0.14	0.31
			1	45	0	0.04	0.04	0.15	0.16
			2	135	0	0.27	-0.27	-0.74	0.83
			3	180	0	0.27	0.00	-0.83	0.87
V	VersaHD	0	4	270	0	0.00	0.37	-0.33	0.50
			5	45	45	0.43	0.14	0.23	0.50
			6	45	90	0.40	0.17	-0.17	0.46
			7	45	270	0.34	-0.06	-0.06	0.35
					Minimum	-1.44	-0.43	-1.50	0.13
					Maximum	1.47	0.92	0.72	1.79
					Median	0.17	0.14	-0.33	0.83
					Average	0.20	0.22	-0.28	0.84
					Standard deviation	0.53	0.38	0.57	0.39
