# Supplementary material for: Spatial accuracy of dose delivery significantly impacts the planning target volume margin in linear accelerator-based intracranial stereotactic radiosurgery
Source: Sci Rep. 2025 Jan 29;15:3608. doi: 10.1038/s41598-025-87769-z (PMC11775166; doi:10.1038/s41598-025-87769-z)
Supplement: Supplementary file 3 — Supplementary Material C [file 41598_2025_87769_MOESM3_ESM.pdf]

Supplement C: Distance between the imaging isocenter and radiation isocenter ($d_{\mathrm{iIC-rIC}}$), determined at each institution. The positive and negative symbols on $d_{\mathrm{iIC-rIC}}$ indicate directions in DICOM reference coordinate systems.

Manufacture	Institution	Linear accelerator	Years of operation	$d_{\mathrm{iIC-rIC}}$ [mm]			
				X	Y	Z	Vector
Varian	A	TrueBeam	10	0.23	0.18	-0.12	0.32
	B	TrueBeamSTx	7	-0.17	-0.03	-0.28	0.33
	C	TrueBeamSTx	7	-0.08	0.11	0.12	0.18
	D	TrueBeam	5	0.12	0.01	-0.19	0.22
	E	TrueBeam	5	-0.08	0.19	-0.03	0.21
	F	TrueBeam	6	-0.21	-0.03	-0.08	0.22
	G	TrueBeamSTx	7	-0.09	-0.04	0.09	0.13
	H	TrueBeamSTx	9	-0.24	-0.3	-0.02	0.38
	I	TrueBeam	7	0.32	-0.19	-0.12	0.39
	J	TrueBeam	6	-0.05	-0.04	0.1	0.12
	K	TrueBeam	1	0.18	0.05	-0.29	0.34
			Minimum	-0.24	-0.30	-0.29	0.12
			Maximum	0.32	0.19	0.12	0.39
			Median	-0.08	-0.03	-0.08	0.22
			Average	-0.01	-0.01	-0.07	0.26
			Standard deviation	0.19	0.15	0.14	0.10
Elekta	L	VersaHD	6	-0.23	0.69	-0.29	0.78
	M	VersaHD	5	0.18	0.11	-0.17	0.27
	N	Infinity	5	0.16	0.76	-0.81	1.13
	O	Synergy	12	0.82	0.39	-0.42	1
	P	Infinity	7	0.06	0.58	-0.71	0.92
	Q	Synergy	12	0.06	1.21	0.51	1.31
	R	Synergy	11	0.02	0.25	-0.54	0.59
	S	VersaHD	4	0.19	0.18	-0.58	0.64
	T	Infinity	9	0.12	0.62	-0.89	1.1
	U	Synergy	7	0.24	-0.13	0.23	0.36
	V	VersaHD	0	0.31	-0.12	-0.33	0.47
			Minimum	-0.23	-0.13	-0.89	0.27
			Maximum	0.82	1.21	0.51	1.31
			Median	0.16	0.39	-0.42	0.78
			Average	0.18	0.41	-0.36	0.78
			Standard deviation	0.26	0.41	0.43	0.34
