# Supplementary material for: Spatial accuracy of dose delivery significantly impacts the planning target volume margin in linear accelerator-based intracranial stereotactic radiosurgery
Source: Sci Rep. 2025 Jan 29;15:3608. doi: 10.1038/s41598-025-87769-z (PMC11775166; doi:10.1038/s41598-025-87769-z)
Supplement: Supplementary file 4 — Supplementary Material D [file 41598_2025_87769_MOESM4_ESM.pdf]

Supplement D: Radius (r) of the smallest sphere that intersects all radiation beams, determined at each institution.

Manufacture	Institution	Linear accelerator	Years of operation	r [mm]
Varian	A	TrueBeam	10	0.43
	B	TrueBeamSTx	7	0.35
	C	TrueBeamSTx	7	0.26
	D	TrueBeam	5	0.41
	E	TrueBeam	5	0.52
	F	TrueBeam	6	0.39
	G	TrueBeamSTx	7	0.31
	H	TrueBeamSTx	9	0.53
	I	TrueBeam	7	0.61
	J	TrueBeam	6	0.31
	K	TrueBeam	1	0.81
			Minimum	0.26
			Maximum	0.81
			Median	0.41
			Average	0.45
			Standard deviation	0.16
Elekta	L	VersaHD	6	0.79
	M	VersaHD	5	0.79
	N	Infinity	5	0.51
	O	Synergy	12	0.81
	P	Infinity	7	0.57
	Q	Synergy	12	0.9
	R	Synergy	11	1.45
	S	VersaHD	4	0.71
	T	Infinity	9	0.62
	U	Synergy	7	0.6
	V	VersaHD	0	0.49
			Minimum	0.49
			Maximum	1.45
			Median	0.71
			Average	0.75
			Standard deviation	0.27
