# Supplementary material for: Spatial accuracy of dose delivery significantly impacts the planning target volume margin in linear accelerator-based intracranial stereotactic radiosurgery
Source: Sci Rep. 2025 Jan 29;15:3608. doi: 10.1038/s41598-025-87769-z (PMC11775166; doi:10.1038/s41598-025-87769-z)
Supplement: Supplementary file 5 — Supplementary Material E [file 41598_2025_87769_MOESM5_ESM.pdf]

Supplement E: Planning target volume margin for each institution, derived from three-dimensional starshot analysis of dose delivery accuracy in this study and from intra-fraction setup errors reported by Ong et al.[26] and Zhang et al.[20]

Setup error (reference)			Ong et al.			Zhang et al.		
Manufacturer	Institution	Years of operation	X	Y	Z	X	Y	Z
Varian	A	10	1.3	1.2	1.2	1.6	1.3	2.0
	B	7	1.2	1.0	1.2	1.6	1.2	2.0
	C	7	1.0	1.0	1.0	1.4	1.2	2.0
	D	5	1.2	1.1	1.2	1.5	1.2	2.0
	E	5	1.3	1.3	1.2	1.6	1.4	2.2
	F	6	1.3	1.0	1.1	1.6	1.2	2.0
	G	7	1.1	1.0	1.1	1.5	1.1	2.0
	H	9	1.4	1.4	1.2	1.8	1.3	2.2
	I	7	1.6	1.4	1.4	1.7	1.4	2.1
	J	6	1.2	1.0	1.1	1.5	1.1	2.0
	K	1	1.7	1.6	1.7	1.9	1.7	2.3
		Minimum	1.0	1.0	1.0	1.4	1.1	2.0
		Maximum	1.7	1.6	1.7	1.9	1.7	2.3
		Average	1.3	1.2	1.2	1.6	1.4	2.1
		Standard deviation	0.2	0.2	0.2	0.1	0.2	0.1
Elekta	L	6	1.7	2.1	1.7	2.1	2.1	2.3
	M	5	1.7	1.6	1.6	1.9	1.7	2.3
	N	5	1.3	1.9	1.9	1.6	1.9	3.0
	O	12	2.3	1.9	1.8	2.3	1.9	2.5
	P	7	1.3	1.8	1.8	1.6	1.8	2.8
	Q	12	1.7	2.8	2.0	2.0	2.8	2.7
	R	11	2.5	2.6	2.8	2.7	2.6	3.4
	S	4	1.6	1.6	1.9	1.8	1.6	2.6
	T	9	1.4	1.9	2.1	1.7	1.9	3.4
	U	7	1.5	1.4	1.4	1.7	1.4	2.3
	V	0	1.4	1.2	1.4	1.6	1.3	2.1
		Minimum	1.3	1.2	1.4	1.6	1.3	2.1
		Maximum	2.5	2.8	2.8	2.7	2.8	3.4
		Average	1.7	1.9	1.9	1.9	1.9	2.7
		Standard deviation	0.4	0.5	0.4	0.4	0.5	0.5
