# Supplementary material for: Spatial accuracy of dose delivery significantly impacts the planning target volume margin in linear accelerator-based intracranial stereotactic radiosurgery
Source: Sci Rep. 2025 Jan 29;15:3608. doi: 10.1038/s41598-025-87769-z (PMC11775166; doi:10.1038/s41598-025-87769-z)
Supplement: Supplementary file 6 — Supplementary Material F [file 41598_2025_87769_MOESM6_ESM.pdf]

Supplement F: Radius (r) of the smallest sphere intersecting all beams, determined for each institution assuming that $d_{\mathrm{iIC-rIC}}$ is zero owing to implementation of rigorous QA procedures and that intra-fraction error caused by treatment couch rotation is zero owing to implementation of a real-time positional monitoring system, such as an advanced image guidance system. Δr represents the reduction in the radius obtained from the results of the linacs without and with the assumed implementation of rigorous QA and an advanced image guidance system.

Manufacture	Institution	Linear accelerator	Years of operation	r [mm]	Δr [mm]
Varian	A	TrueBeam	10	0.21	0.22
	B	TrueBeamSTx	7	0.27	0.08
	C	TrueBeamSTx	7	0.25	0.01
	D	TrueBeam	5	0.25	0.16
	E	TrueBeam	5	0.23	0.29
	F	TrueBeam	6	0.30	0.09
	G	TrueBeamSTx	7	0.30	0.01
	H	TrueBeamSTx	9	0.32	0.21
	I	TrueBeam	7	0.29	0.32
	J	TrueBeam	6	0.23	0.08
	K	TrueBeam	1	0.28	0.53
			Minimum	0.21	0.05
			Maximum	0.32	0.49
			Median	0.27	0.14
			Average	0.27	0.18
			Standard deviation	0.04	0.13
Elekta	L	VersaHD	6	0.63	0.16
	M	VersaHD	5	0.76	0.03
	N	Infinity	5	0.50	0.01
	O	Synergy	12	0.62	0.19
	P	Infinity	7	0.57	0.00
	Q	Synergy	12	0.63	0.27
	R	Synergy	11	0.64	0.81
	S	VersaHD	4	0.35	0.36
	T	Infinity	9	0.62	0.00
	U	Synergy	7	0.60	0.00
	V	VersaHD	0	0.49	0.00
			Minimum	0.35	0.14
			Maximum	0.76	0.69
			Median	0.62	0.09
			Average	0.58	0.17
			Standard deviation	0.11	0.16
